# Supplementary material for: Asynchronous magnetic resonance elastography: Shear wave speed reconstruction using noise correlation of incoherent waves
Source: Magn Reson Med. 2022 Oct 27;89(3):990–1001. doi: 10.1002/mrm.29502 (PMC9792433; doi:10.1002/mrm.29502)
Supplement: Supplementary file 1 — DATA S1 MATLAB code used to generate the numerical simulations of Figure 1 [file MRM-89-990-s002.zip › k-Wave/helpfiles/example_ivp_3D_simulation.html]

Simulations In Three Dimensions Example (k-Wave)


# Simulations In Three Dimensions Example

This example provides a simple demonstration of using k-Wave for the simulation and detection of the pressure field generated by an initial pressure distribution within a three-dimensional heterogeneous propagation medium. It builds on the Homogeneous Propagation Medium and Heterogeneous Propagation Medium examples.

- Open the file in the MATLAB Editor
- Run the file in MATLAB

## Contents

- Creating the k-space grid and defining the medium properties
- Defining the initial pressure distribution
- Defining the sensor mask
- Running the simulation
- A note on Cartesian sensor masks

## Creating the k-space grid and defining the medium properties

Simulations in three-dimensions are performed in an analogous fashion to those in one and two dimensions. The medium discretisation is again performed by `kWaveGrid` with two additional inputs for the z-dimension. Similarly, matrices for the medium properties also have an extra dimension. The matrix indexing convention (x, y, z) is an extension to that used in two-dimensions (x, y) where matrix row and column data correspond to the x and y axes, respectively.

```
% create the computational grid
Nx = 64;            % number of grid points in the x direction
Ny = 64;            % number of grid points in the y direction
Nz = 64;            % number of grid points in the z direction
dx = 0.1e-3;        % grid point spacing in the x direction [m]
dy = 0.1e-3;        % grid point spacing in the y direction [m]
dz = 0.1e-3;        % grid point spacing in the z direction [m]
kgrid = kWaveGrid(Nx, dx, Ny, dy, Nz, dz);

% define the properties of the propagation medium
medium.sound_speed = 1500 * ones(Nx, Ny, Nz);	% [m/s]
medium.sound_speed(1:Nx/2, :, :) = 1800;        % [m/s]
medium.density = 1000 * ones(Nx, Ny, Nz);       % [kg/m^3]
medium.density(:, Ny/4:end, :) = 1200;          % [kg/m^3]
```

## Defining the initial pressure distribution

As in one and two dimensions, the initial pressure distribution is defined as a matrix of arbitrary numbers the same size as the computational grid. Here `makeBall` is used to create an initial pressure distribution of two small filled balls with different diameters.

```
% create initial pressure distribution using makeBall
ball_magnitude = 10;    % [Pa]
ball_x_pos = 38;        % [grid points]
ball_y_pos = 32;        % [grid points]
ball_z_pos = 32;        % [grid points]
ball_radius = 5;        % [grid points]
ball_1 = ball_magnitude * makeBall(Nx, Ny, Nz, ball_x_pos, ball_y_pos, ball_z_pos, ball_radius);

ball_magnitude = 10;    % [Pa]
ball_x_pos = 20;        % [grid points]
ball_y_pos = 20;        % [grid points]
ball_z_pos = 20;        % [grid points]
ball_radius = 3;        % [grid points]
ball_2 = ball_magnitude * makeBall(Nx, Ny, Nz, ball_x_pos, ball_y_pos, ball_z_pos, ball_radius);

source.p0 = ball_1 + ball_2;
```

## Defining the sensor mask

Again the sensor mask can be given as a list of Cartesian coordinates, a binary mask, or the grid coordinates of two opposing corners of a cuboid. Several functions for creating three-dimensional shapes are included in the k-Wave toolbox. For example, `makeSphere` which returns a binary map of single pixel spherical shell created using the midpoint circle algorithm, and `makeCartSphere` which returns the Cartesian location of an evenly distributed array of an arbitrary number of points on the surface of a sphere. In this example, a linear sensor array is explicitly created.

```
% define a series of Cartesian points to collect the data
x = (-22:2:22) * dx;          % [m]
y = 22 * dy * ones(size(x));    % [m]
z = (-22:2:22) * dz;          % [m]
sensor.mask = [x; y; z];
```

A visualisation of the initial pressure distribution and the sensor mask using `cart2grid` and `voxelPlot` is given below.


## Running the simulation

The computation is invoked by calling `kspaceFirstOrder3D` with the inputs defined above. By default, a visualisation of the wave-field in the horizontal, median, and frontal planes are displayed, along with a status bar.

```
% input arguments
input_args = {'PlotLayout', true, 'PlotPML', false, ...
    'DataCast', 'single', 'CartInterp', 'nearest'};

% run the simulation
sensor_data = kspaceFirstOrder3D(kgrid, medium, source, sensor, input_args{:});
```

By setting the optional input parameter `'PlotLayout'` to `true`, a plot of the initial pressure, and sound speed and density (if heterogenous) are produced. To remove the PML from the display, the optional input `'PlotPML'` is also set to `false`.


As the function runs, status updates and computational parameters are printed to the command line. By setting the optional input `'DataCast'` to `'single'`, the computational time is decreased (see the Optimising k-Wave Performance Example for more details).

```
Running k-Wave simulation...
  start time: 13-Apr-2017 22:02:40
  reference sound speed: 1800m/s
  dt: 16.6667ns, t_end: 7.3833us, time steps: 444
  input grid size: 64 by 64 by 64 grid points (6.4 by 6.4 by 6.4mm)
  maximum supported frequency: 7.5MHz
  smoothing p0 distribution...
  casting variables to single type...
  precomputation completed in 2.7428s
  starting time loop...
  estimated simulation time 15.6821s...
  simulation completed in 16.4262s
  reordering Cartesian measurement data...
  total computation time 19.286s
```

When the time loop has completed, the function returns the recorded time series at each of sensor locations defined by `sensor.mask`. The ordering is again dependent on whether a Cartesian or binary sensor mask is used. A visualisation is given below.


## A note on Cartesian sensor masks

If a Cartesian sensor mask is used, the values of the acoustic field at the sensor points are obtained at each time step using interpolation. The interpolation mode can be changed by setting the optional input parameter `'CartInterp'` to `'linear'` (the default) or `'nearest'` (used in this example). During the simulation, there is only a small performance difference between using Cartesian and binary sensor masks. However, if a Cartesian sensor mask with linear interpolation is used, the calculation of the triangulation points can significantly lengthen the precomputation time, particularly for 3D simulations (see the output below for the same example run with `'CartInterp'` set to `'linear'`). A Cartesian sensor mask can be converted to a binary sensor mask (and vice versa) using the functions `cart2grid` and `grid2cart`.

```
Running k-Wave simulation...
  start time: 18-Oct-2011 10:08:44
  reference sound speed: 1800m/s
  dt: 16.6667ns, t_end: 6.15us, time steps: 370
  input grid size: 64 by 64 by 64 grid points (6.4 by 6.4 by 6.4mm)
  maximum supported frequency: 7.5MHz
  smoothing p0 distribution...
  calculating Delaunay triangulation...
  precomputation completed in 25.1272s
  starting time loop...
  estimated simulation time 44.8s...
  simulation completed in 50.4407s
  total computation time 1min 15.598s
```

In both two and three dimensional simulations, if the optional input `'CartInterp'` is set to `'nearest'`, the Cartesian points are internally mapped onto a binary grid. If more than one Cartesian sensor point maps to the same location, the duplicated points are discarded. Consequently, if the Cartesian sensor mask has a dense array of points, the number of returned time-series may not correspond to the original number of Cartesian sensor points. Details of discarded duplicate points are printed to the command line. This situation can be avoided by increasing the resolution of the computational grid, or by reducing the packing of the Cartesian sensor points.
